# “Birthday-Banding” as a Strategy to Moderate the Relative Age Effect: A Case Study Into the England Squash Talent Pathway

**DOI:** 10.3389/fspor.2020.573890

**Published:** 2020-11-17

**Authors:** Adam L. Kelly, Daniel T. Jackson, Josh J. Taylor, Mark A. Jeffreys, Jennifer Turnnidge

**Affiliations:** ^1^Department of Sport and Exercise, Research Centre for Life and Sport Sciences (CLaSS), Birmingham City University, Birmingham, United Kingdom; ^2^Independent Researcher, National Squash Centre, Manchester, United Kingdom; ^3^PLAYS Research Group, School of Kinesiology and Health Studies, Queen's University, Kingston, ON, Canada

**Keywords:** athlete development, talent development, talent identification, skill acquisition, expertise, RAE, youth sport, bio-banding

## Abstract

The relative age effect (RAE) is almost pervasive throughout youth sports, whereby relatively older athletes are consistently overrepresented compared to their relatively younger peers. Although researchers regularly cite the need for sports programs to incorporate strategies to moderate the RAE, organizational structures often continue to adopt a one-dimensional (bi)annual-age group approach. In an effort to combat this issue, England Squash implemented a “birthday-banding” strategy in its talent pathway, whereby young athletes move up to their next age group on their birthday, with the aim to remove particular selection time points and fixed chronological bandings. Thus, the purpose of this study was to examine the potential effects of the birthday-banding strategy on birth quarter (BQ) distributions throughout the England Squash talent pathway. Three mixed-gender groups were populated and analyzed: (a) ASPIRE athletes (*n* = 250), (b) Development and Potential athletes (*n* = 52), and (c) Senior team and Academy athletes (*n* = 26). Chi-square analysis and odds ratios were used to test BQ distributions against national norms and between quartiles, respectively. Results reveal no significant difference between BQ distributions within all three groups (*P* > 0.05). In contrast to most studies examining the RAE within athlete development settings, there appears to be no RAE throughout the England Squash talent pathway. These findings suggest that the birthday-banding strategy may be a useful tool to moderate RAE in youth sports.

## Introduction

More than 20 million people across 185 countries regularly participate in squash (US Squash, 2019); thus, reaching the highest levels of performance can be extremely competitive. The aim of a talent pathway is to recruit young athletes with the prospect of advancing into experts at the senior professional level by providing them with the most appropriate learning environment to achieve their potential (Kelly et al., 2018). In an effort to fulfill this aim, sport organizations have emphasized the importance of identifying early predictors for long-term attainment so that the most highly talented youth athletes receive continued support from a young age (Stratton et al., 2004). However, the complex nature of the talent development process suggests that the application of early predictors is often flawed and subject to selection biases (Baker et al., 2003).

One such bias is the influence of selection and progression through fixed annual birthdate distribution—known as the relative age effect (RAE; Barnsley et al., 1985). Research consistently highlights that youth athletes born earlier in the selection year relative to a predetermined cutoff date (e.g., September 1 to August 31) are often overrepresented within talent development pathways compared to those born later in the same selection year (Cobley et al., 2009). Indeed, the RAE is a global phenomenon, indicating that cultural factors (e.g., nationality, traditional preferences, socioeconomic circumstance) are potentially extraneous and are independent of specific cutoff dates (e.g., Helsen et al., 2005; Nakata and Sakamoto, 2013; Turnnidge et al., 2014; Cobley et al., 2018). In addition, the RAE is almost ubiquitous throughout talent development pathways in youth sport when (bi)annual age grouping is adopted although in certain sports it may be more prevalent (e.g., soccer) than others (gymnastics; Smith et al., 2018).

Although squash appears to be an unexplored sport among RAE literature, other racquet sports (e.g., badminton, table tennis, tennis) are consistent with the findings of an overrepresentation of players born in the first half of the year compared to their later born age group equivalents at the youth level (e.g., Ulbricht et al., 2015; Romann et al., 2018; Faber et al., 2020). For instance, previous studies document a skewed birthdate distribution in youth tennis, whereby a higher number of athletes involved in talent development programs were born in the first half of the selection year (e.g., values ranging from 60 to 86%) compared to the second half (Dudink, 1994; Filipcic, 2001; Edgar and O'Donoghue, 2005; Loffing et al., 2010). As an example, Ulbricht et al. (2015) find a similar trend throughout the German Tennis Federation male talent pathway (U12 to U18) with RAEs more prevalent at higher competition levels. For instance, more selected players were born in the first half of the year (compared with normative values) for national (70.2%) and regional (65.1%) players. Interestingly, they find little evidence to suggest an RAE in senior ranked representatives (56% born in the first half of the year). Thus, it is possible that, during the transition from the elite youth level to senior professional status, a greater number of relatively older athletes are more likely to drop out of talent pathways, which is also reported in various other sports (e.g., Cobley et al., 2008; Baker et al., 2010; Gil et al., 2020).

Sticking with the theme of racquet sports, Faber et al. (2019) found an RAE among French table tennis players aged 14–21 years. Similarly, during their investigation into the Swiss national talent development program, Romann et al. (2018) reveal that both tennis and badminton pathways had a pronounced RAE, again favoring those born in the first half of the year. Although the RAE is consistently found at youth levels, findings at senior levels are equivocal. For instance, Romann et al. (2018) and Faber et al. (2019) find no RAE among their senior tennis and table tennis cohorts, respectively. Correspondingly, Nakata and Sakamoto (2013) find no significant differences in the birth quartile distribution of their senior Japanese male badminton population. These conflicting findings underscore the importance of exploring the prevalence of RAEs at different stages of development.

It is evident that there is a complicated relationship between the month in which athletes are born, their opportunities to be selected into a talent pathway, and their likelihood of successfully transitioning from such a program (Kelly et al., 2020). Although there is an extensive body of RAE research over the last three decades, questions still remain concerning the organizational structures that underpin these relationships (Cobley et al., 2019). The need to examine these structures is emphasized by a growing body of literature exploring potential strategies for mitigating RAEs in youth sports programs (Romann and Cobley, 2015; Mann and van Ginneken, 2017; Cobley et al., 2019; Webdale et al., 2019). In order to gain a deeper understanding of the RAE, it is important to recognize that it represents a by-product of sports organizations' policies regarding grouping athletes by chronological age. Although such policies are often intended to promote developmentally appropriate levels of challenge and create fair competition, it is evident that these policies can have unintended consequences (Baxter-Jones, 1995). As such, it is worthwhile to explore the different types of grouping strategies that can be used within organizations as well as the potential implications of these strategies on athletes' developmental trajectories.

In an attempt to combat the RAE due to a fixed chronological age group approach, England Squash implemented a *birthday-banding* strategy within their talent pathway 7 years ago. Birthday-banding refers to the organizational policy whereby young athletes move up to their next birthdate group on their birthday with the aim of removing particular selection time points and fixed chronological age groups. As an example, a U13 player would move up to the U14 age group on their birthday and remain in that age group until their following birthday. As such, recruitment remains continual to ensure there is an equal opportunity for all players to be selected during the entire selection year. Although this strategy has been implemented in practice, the relation between the birthday-banding strategy and birth quartile distributions has yet to be empirically evaluated. Thus, the aim of this study is to examine birth quartile distributions against normative values within the England Squash talent pathway. Drawing upon existing literature in racquet sports, it was hypothesized that there would be an RAE within the youth cohorts but not among the adult cohorts.

## Methods

### Sample and Design

**Figure 1:**
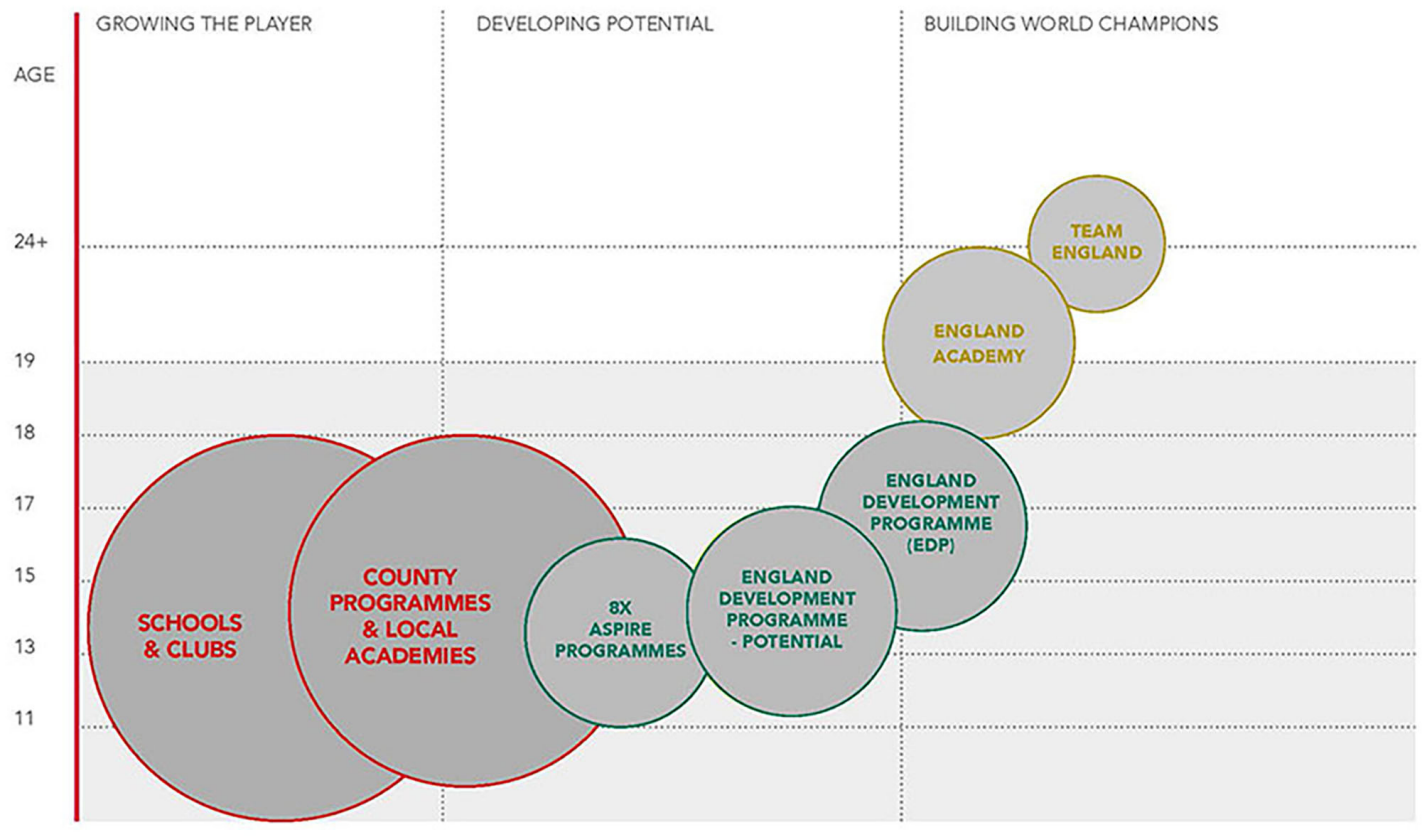
The England Squash talent pathway.

A combined total of 328 participants (male = 188, female = 126) from the England Squash talent pathway are included in this study. Following two grassroots entry levels (schools and clubs and county programs and local academies), the talent pathway comprises five selection levels within a progressive structure (see Figure 1): (a) ASPIRE (*n* = 250; *M*_age_ = 13.9 ± 2.1 years; male = 157, female = 93), (b) Potential (*n* = 27; *M*_age_ = 13.5 ± 1.4 years; male = 14, female = 13), (c) Development (*n* = 26; *M*_age_ = 17.1 ± 1.3 years; male = 15, female = 10), (d) Academy (*n* = 12; *M*_age_ = 20.1 ± 2.3 years; male = 8, female = 4), and (e) Senior team (*n* = 14; *M*_age_ = 29.8 ± 4.3 years; male = 8, female = 6). ASPIRE acts as the first stepping-stone onto the England Squash talent pathway, which offers the most promising young players an environment to develop within each English region. This leads into the Potential cohort, which is focused on providing the first national-level squad for the younger and developing talent in the country. This develops and feeds the pool of players for the Development cohort, which is for those who wish to continue their progression in the sport to a world-class level toward the Academy and Senior team (England Squash, 2020).

Training time varies across the five selection levels: (a) ASPIRE = 3–6 h/week; (b) Potential = 5–10 h/week; (c) Development = 7–14 h/week; (d) Academy = 15–20 h/week; and (e) Senior team = 15–20 h/week. To create a more accurate representation of participation in the pathway and because the sample sizes are limited, ASPIRE athletes were analyzed on their own, and Development and Potential (*n* = 53) and Senior team and Academy (*n* = 26) are grouped together for analysis to create three cohorts. Because mixed-gender training and competition is common practice throughout the England Squash talent pathway (and because the sample sizes are limited), male and female athletes were analyzed together within the three cohorts. To offer a comparison between genders, the male and female combined cohorts were also analyzed. This study received ethical approval from the lead author's institution.

### Procedures

The 12 months of the year were divided into four birth quarters (BQs), conforming to the strategy used to examine the RAE in other UK populated studies (e.g., Helsen et al., 2005). In line with the chronological age grouping system applied in the UK, September was classified as *month 1* descending to August as *month 12*. To conform with previous studies of a similar design (e.g., Kelly et al., 2020), athletes were assigned a BQ based on their selection year. These were subsequently compared to the expected distributions from the calculated average national live births in England and Wales from 1999 to 2008 to provide a similar age of birth to that of the sample (Office for National Statistics, 2015).

### Data Analysis

Chi-square (χ^2^) goodness of fit analysis was used to compare BQ distributions in the sample against population values (Office for National Statistics, 2015) following procedures outlined by McHugh (2013). As this test does not reveal the magnitude of difference between BQ distributions for significant chi-square outputs, Cramer's V was also used. The Cramer's V was interpreted as per conventional thresholds for correlation: a value of 0.06 or more would indicate a small effect size, 0.17 or more would indicate a medium effect size, and 0.29 or more would indicate a large effect size (Cohen, 1988). Odds ratios (ORs) and 95% confidence intervals (CIs) were used to compare BQs for observed and expected distributions. For all the tests, results were considered statistically significant when *P* < 0.05. All statistical analyses were conducted using IBM SPSS Statistics Version 24.

## Results

**Figure 2:**
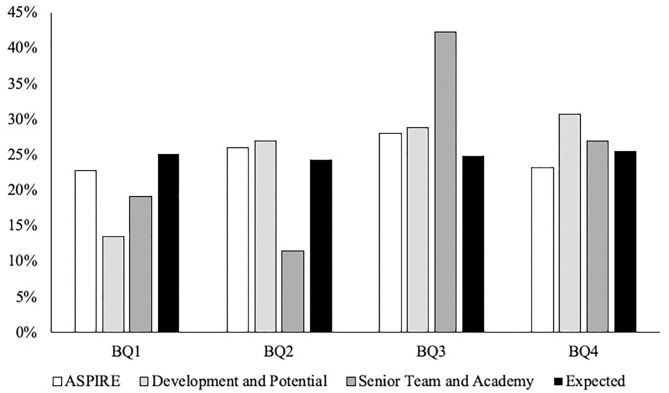
The distribution of BQs in athlete's respective cohort and expected distribution from national norms (Office for National Statistics, 2015).

**Figure 3:**
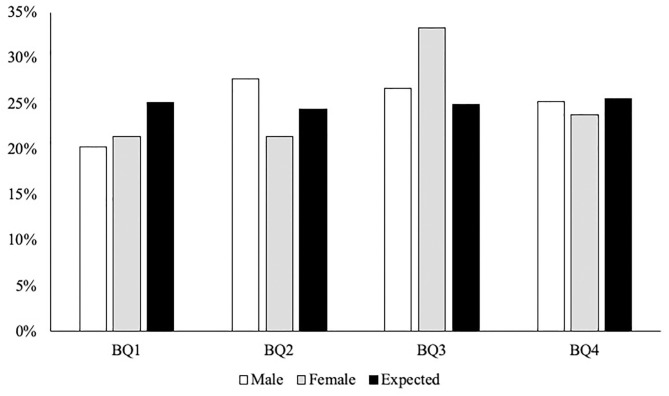
The distribution of BQs in male and female combined cohorts and expected distribution of national norms (Office for National Statistics, 2015).

In line with the England Squash talent pathway selection levels, results are presented in ascending order: (a) ASPIRE, (b) Development and Potential, and (c) Senior team and Academy. Total combined male and female results are then also presented. First, there was no significant difference in the ASPIRE BQ distributions compared to national norms [$\chi_{\left(df=3\right)}^{2}$ = 2.292, *P* = 0.514, *V* = 0.07; see Figure 2]. There were also no significant ORs found between BQ distributions. Second, there was no significant difference in the Development and Potential BQ distributions compared to national norms [$\chi_{\left(df=3\right)}^{2}$ = 3.872, *P* = 0.238, *V* = 0.19; see Figure 2]. There were also no significant ORs found between BQ distributions. Third, there was no significant difference in the Senior team and Academy BQ distributions compared to national norms [$\chi_{\left(df=3\right)}^{2}$ = 5.290, *P* = 0.152, *V* = 0.32; see Figure 2]. There were also no significant ORs found between BQ distributions. Finally, there was no significant difference in the male combined cohort BQ distributions compared to national norms [$\chi_{\left(df=3\right)}^{2}$ = 5.290, *P* = 0.152, *V* = 0.32; see Figure 3]. There were also no significant ORs found between BQ distributions. Furthermore, there was no significant difference in the female combined cohort BQ distributions compared to national norms [$\chi_{\left(df=3\right)}^{2}$ = 5.290, *P* = 0.152, *V* = 0.32; see Figure 3]. There were also no significant ORs found between BQ distributions. The descriptive statistics for all three cohorts as well as the total combined male and female cohorts are presented in Table 1.

**Table 1:** The distributions of BQs with chi-square, Cramer's V, and OR analysis.

**Quartile distributions (ONS)**	**BQ1 (25.12%)**	**BQ2 (24.39%)**	**BQ3 (24.93%)**	**BQ4 (25.56%)**	**Total**	**χ^2^ (*df* = 3)**	***P***	**Cramer's V**	**BQ1 vs. BQ4 OR (95% CI)**
ASPIRE	57 (62.80)	65 (60.98)	70 (62.32)	58 (63.90)	250	2.292	0.514	0.07	1.00 (0.604; 1.657)
Development and potential	7 (13.06)	14 (12.68)	15 (12.96)	16 (13.29)	52	3.822	0.281	0.19	0.45 (0.138; 1.436)
Senior team and academy	5 (6.53)	3 (6.34)	11 (6.48)	7 (6.65)	26	5.290	0.152	0.32	0.73 (0.150; 3.514)
Male combined cohort	41 (50.74)	56 (49.27)	54 (50.36)	51 (51.63)	202	3.061	0.382	0.09	0.82 (0.465; 1.439)
Female combined cohort	27 (31.65)	27 (30.73)	42 (31.41)	30 (32.21)	126	4.857	0.183	0.14	0.91 (0.448; 1.872)

## Discussion

The aim of the current study was to examine RAEs within the English Squash talent pathway. This sports setting provides a unique context to examine RAEs because they group players into *birthday-bands* rather than traditional (bi)annual-age groups. Findings reveal that there were no observed RAEs within the England Squash talent pathway across all cohorts. In recognizing that numerous factors may contribute to the lack of an observed RAE, the following sections explore potential explanations for these findings and discuss the limitations and future directions of this research.

Given the pervasive nature of RAEs in youth sports (Cobley et al., 2009; Smith et al., 2018), it is interesting to consider the lack of RAEs within the squash setting. There are several possible explanations for this insignificant RAE. First, previous studies have found inconsistent RAEs within senior racquet sport contexts (e.g., Ulbricht et al., 2015; Romann et al., 2018; Faber et al., 2019). In line with the current study's findings, previous research in racquet sports, such as badminton (e.g., Nakata and Sakamoto, 2013), table tennis (e.g., Faber et al., 2019), and tennis (e.g., Ulbricht et al., 2015) finds no significant differences in BQ distributions at a senior level. Conversely, however, the present study finds no RAEs at youth levels, whereas RAEs are well-established in other youth racquet sports, including badminton (e.g., Romann et al., 2018), table tennis (e.g., Faber et al., 2020), and tennis (e.g., Loffing et al., 2010). Due to these conflicting findings, it is worthwhile to further explore the organizational and sociocultural structures that may be influencing the occurrence of RAEs at the youth level in other sporting contexts.

Second, it is possible to suggest that the use of birthday-banding for group players within the England Squash talent pathway may contribute to the insignificant RAEs. As discussed, birthday-banding offers athletes the opportunity to consistently shift between being the relatively oldest and the relatively youngest player based on their individual age throughout development. As a result, this strategy may be more accurate in capturing the dynamic nature of the athlete development process (Collins and MacNamara, 2012; Collins et al., 2016). More specifically, rather than competing in *fixed* (bi)annual-age groups (i.e., being relatively older or relatively younger throughout development), the birthday-banding strategy facilitates more diverse experiences (i.e., being both relatively older *and* relatively younger throughout development). Nevertheless, it is important to recognize that there may be other contributing factors that influence the equal BQ distributions throughout the selection levels (e.g., social context, competition level, and the number of active participants; Musch and Grondin, 2001). Thus, further research with a larger sample size in a different sports context is required before results are generalizable.

In order to understand the potential benefits of this strategy, literature examining mixed-age and play can be drawn upon. Evidence exists to suggest that older and younger participants can draw unique benefits from playing with each other. For example, relatively older athletes can experience opportunities for leadership and helping of younger peers (Côté et al., 2007; Jarvis, 2007). On the other hand, relatively younger athletes may benefit from the opportunity to hone their skills and compete against older teammates (Gibbs et al., 2012; Kelly et al., 2020). Because birthday-banding enables this shift between being relatively older and younger among one's peers, it may offer athletes more diverse developmental experiences along the talent pathway. Further, the potential for increased diversity extends beyond athletes' relative age within their peer group. For example, athletes may also have increased opportunities to interact with different coaches as well as engage in different types of activities. Collectively, these diverse experiences may facilitate athletes' immediate, short-, and long-term developmental outcomes (Côté et al., 2014).

Another potential benefit for the birthday-banding approach may be that it enables athletes to experience different types of social comparison environments. According to Wood and Wilson (2003), social comparison theory suggests that athletes rely on peers and teammates as a frame of reference to compare themselves, which is used to build self-perceptions, such as competence and identity. Applied to the birthday-banding context, it is possible that opportunities to be the relatively older athlete in the group provide a positive setting for athletes to compare themselves with others. In doing so, birthday-banding may enable a greater number of athletes to experience being the top performer within their group. On the other hand, when athletes move to the older age groups, they may be required to reevaluate their self-perceptions in relation to their new peer group (Goldman et al., under review). By amplifying opportunities for social comparison, the birthday-banding approach may encourage youth to develop a more dynamic and resilient sense of self throughout development. Given the limited body of literature on this topic, further research is warranted to substantiate this claim.

The origins of RAEs must also be considered when interpreting the insignificant BQ distributions throughout each cohort. For instance, gender (i.e., male vs. female), sport type (i.e., team vs. individual), and competition level (e.g., recreational participation vs. talent development) may play an important role in constructing RAEs (Cobley et al., 2009). First, previous research documents stronger RAEs in males when compared to females (e.g., Brustio et al., 2019). However, because the current findings reveal no differences between BQ distributions in both male and female cohorts, birthday-banding may be a useful grouping strategy to moderate RAEs within both genders. Second, the individual nature of squash may be a contributing factor toward the insignificant BQ distributions. Previous meta-analytic findings from Smith et al. (2018) reveal female team sports are associated with higher RAE estimates when compared to individual sports (ORs = team 1.33 vs. individual 1.18). This may be due to the comparisons between team sports athletes that occur during competition and are often subjective in nature, thus potentially placing greater emphasis on physiological differences (Baker et al., 2014). When considering the birthday-banding approach in a team sport context, however, it may prove difficult to implement due to squad requirements and additional administrative duties. Finally, previous studies document higher RAEs with increased competition level. Because the England Squash talent pathway is the highest competition level within the sport, it is plausible to suggest that it may be at greater risk of RAEs when compared to recreational participation. Thus, in light of the insignificant BQ distributions, the current findings suggest that birthday-banding may be a particularly robust grouping approach to moderate RAEs at the highest competition levels.

### Alternative Organizational Structures

To understand the findings of the present study, it is also worthwhile to examine the literature on other strategies designed to moderate RAEs. For instance, Romann and Cobley (2015) and Cobley et al. (2019) have devised a method named *corrective adjustments* as a solution to remove RAEs in timed sports, such as athletics and swimming. Corrective adjustments refer to a process in which regression equations are applied through birthdate distribution and raw performance times with the disseminations of performance levels subsequently reexamined for greater relative age equality. Although this strategy may be appropriate for timed sports, it may be difficult to implement within racquet sports, such as squash, due to the interactive nature and scoring processes.

One strategy that may be particularly relevant for the present study is the *bio-banding* approach (Malina et al., 2019). Bio-banding involves grouping athletes based on their biological age as determined by their individual maturation status. This approach appears advantageous because it reduces inequality in competition that occurs due to growth and maturation differences between athletes in the same (bi)annual-age group (Malina et al., 2015). More specifically, when athletes with larger body types compete against each other, they have been shown to rely less on their size and more on their skill to succeed (Cumming et al., 2018). At the same time, when athletes with smaller body types compete against each other, they may be exposed to more manageable levels of challenge (Malina et al., 2015; Bradley et al., 2019). Interpreting these findings in light of the current study, it is possible that both bio-banding and birthday-banding may provide youth with competitive experiences that are tailored to individual developmental trajectories. However, Cumming et al. (2017) explicitly state this tool is designed to moderate maturation biases and should not be confused with mitigating RAEs as current research suggests only a weak-to-moderate proxy of maturation in youth athletes. In contrast though, Smith et al. (2018) meta-analysis reveals female weight-categorized sport types are generally not associated with RAEs, suggesting anthropometric bands may indeed be a useful strategy to moderate RAEs in youth sports. As such, further research exploring the influence of these strategies on RAEs in various youth sport contexts is warranted.

One of the potential advantages of the birthday-banding approach is that it may remove the effects of selection biases. Whereas, previous studies focus on interventions designed to target selection biases, birthday-banding may eliminate the need for such interventions. For example, researchers have proposed methods, such as establishing *selection quotas* (i.e., organizations are required to select a minimum number of athletes from each BQ; Kyle et al., 2019) or applying *age-ordered shirt numbering* systems (Mann and van Ginneken, 2017), which may be useful strategies for minimizing selection biases within (bi)annual-age groups. Within the birthday-banding context, such strategies may be less relevant as young players are not restricted to a fixed group or BQ throughout the year or during their development, respectively. Rather, by using an athlete's birthday as a proxy indicator of their readiness to progress to higher competition levels, this approach may more accurately capture the dynamic nature of athletes' developmental needs, which may change throughout the course of a competitive season.

Given the limited body of research on youth grouping in sports, it may also be worthwhile to examine grouping research within educational contexts, which has similarly explored issues relating to the effects of grouping strategies on academic achievement (Thompson et al., 2004). Two forms of grouping strategies that have received considerable attention in education include (a) *achievement grouping*, which refers to a constellation of processes whereby students of similar achievement levels are placed together for the purpose of tailoring educational experiences to student needs, and (b) *acceleration*, which involves students who progress through school faster in relation to their peers (i.e., *skipping a grade*; Neihart, 2007; Steenbergen-Hu et al., 2016). Because the aim of these strategies is to provide youth with developmentally appropriate learning opportunities, they share similarities with the birthday-banding approach. It may, thus, be useful for sport researchers to draw upon this literature to integrate a wider range of concepts and theories to inform their grouping strategies. In doing so, researchers can expand beyond the silo of sport science to improve the quality and equity of talent pathways.

### Limitations and Future Directions

The authors acknowledge that it is difficult to fully determine birthday-banding as the solitary cause of the equal BQ distributions throughout the England Squash talent pathway. Because athlete development is a complex and multidimensional process (e.g., Kelly and Williams, 2020), it is important to appreciate that there may be other contributing factors that have influenced the balanced BQ distributions throughout the selection levels (e.g., social context, competition level, and the number of active participants; Musch and Grondin, 2001). As an example, it has been previously documented that, during a comparison of different youth sports, those with greater popularity often comprise a stronger RAE (Lupo et al., 2019). As such, it may be argued that the lower participation levels of squash compared to other sports in England (e.g., soccer) may result in less difficulty during the selection process, and thus, a weaker RAE occurs. Furthermore, although the small sample limits the statistical power of the cohorts, it provides an accurate and comprehensive representation of the England Squash talent pathway, which offers a contextualized assessment of RAEs within a real-world setting. It is encouraged that future research replicate this study design within a larger cohort to ensure the birthday-banding strategy is generalizable. In addition, although male and female combined cohorts were analyzed to explore the overall gender differences, due to the small sample size and to ensure an appropriate level of statistical rigor was applied, gender comparisons within each selection level were not included as part of the analysis. However, because mixed-gender training and competition regularly operate throughout the England Squash talent pathway, it represents an accurate representation of each cohort. Nevertheless, exploring additional gender differences represents a fruitful avenue for future research.

The current findings offer an insight into the potential outcomes of birthday-banding. However, numerous questions remain to understand how this approach is viewed and experienced by key stakeholders. As such, investigations with athletes, coaches, and peers may extend beyond our current understanding of the lived experience of participating in sport settings that adopt this grouping approach. Qualitative and observational methodologies may be instrumental in addressing this gap. Indeed, studies employing these methods may shed light on the potential mechanisms underpinning the association between birthday-banding and RAEs. Furthermore, it is important to acknowledge that this study only assessed birthday-banding at one point in time. As such, future studies can build upon this research by using longitudinal designs to investigate the effects of the birthday-banding approach throughout athletes' developmental trajectories.

## Conclusion

In sum, the findings from this current study are consistent with other RAE research among racquet sports (e.g., badminton, table tennis, tennis) at adulthood (i.e., Senior team and Academy selection levels), whereby no significant difference in BQ distributions were revealed. However, while exploring the youth selection levels (i.e., ASPIRE, Development, and Potential), the results from this current study are contrary to existing RAE literature in other racquet sports, whereby no significant differences in BQ distributions were apparent. The authors introduce the concept of birthday-banding, which has been widely adopted by the England Squash talent pathway as a grouping strategy with the main purpose of moderating the RAE. Although it is difficult to interpret birthday-banding as the single cause for there being no RAEs throughout the England Squash talent pathway, it can be suggested that it is more representative of the dynamic nature of the athlete development process when compared to fixed (bi)annual-age grouping. Coaches and practitioners working in youth sports are encouraged to challenge traditional age group structures to help eliminate annual-age biases throughout athlete development systems.

## Data Availability Statement

The raw data supporting the conclusions of this article will be made available by the authors, without undue reservation.

## Ethics Statement

The studies involving human participants were reviewed and approved by Birmingham City University Health, Education and Life Sciences Faculty Academic Ethics Committee. Written informed consent from the participants' legal guardian/next of kin was not required to participate in this study in accordance with the national legislation and the institutional requirements.

## Author Contributions

DJ, JJT, and MJ primarily focused on the Methods and Results sections, whereas AK and JT contributed more to the Introduction, Discussion, and Conclusion. All authors were involved with compiling the data, as well as writing the full manuscript.

## Conflict of Interest

The authors declare that the research was conducted in the absence of any commercial or financial relationships that could be construed as a potential conflict of interest.
